# Laser-wakefield accelerators as hard x-ray sources for 3D medical imaging of human bone

**DOI:** 10.1038/srep13244

**Published:** 2015-08-18

**Authors:** J. M. Cole, J. C. Wood, N. C. Lopes, K. Poder, R. L. Abel, S. Alatabi, J. S. J. Bryant, A. Jin, S. Kneip, K. Mecseki, D. R. Symes, S. P. D. Mangles, Z. Najmudin

**Affiliations:** 1 The John Adams Institute for Accelerator Science, Blackett Laboratory, Imperial College London, London SW7 2BZ, UK; 2 GoLP, Instituto de Plasmas e Fusão Nuclear, Instituto Superior Técnico, Universidade de Lisboa, 1049-001, Portugal; 3 Department of Surgery and Cancer, MSk Laboratory, Charing Cross Hospital, Imperial College London, London W6 8RF, UK; 4 Department of Mechanical Engineering, City and Guilds Building, Imperial College London, London SW7 2AZ, UK; 5 Central Laser Facility, Rutherford Appleton Laboratory, Didcot OX11 0QX, UK

## Abstract

A bright *μ*m-sized source of *hard* synchrotron x-rays (critical energy *E*_*crit*_ > 30 keV) based on the betatron oscillations of laser wakefield accelerated electrons has been developed. The potential of this source for medical imaging was demonstrated by performing micro-computed tomography of a human femoral trabecular bone sample, allowing full 3D reconstruction to a resolution below 50 *μ*m. The use of a 1 cm long wakefield accelerator means that the length of the beamline (excluding the laser) is dominated by the x-ray imaging distances rather than the electron acceleration distances. The source possesses high peak brightness, which allows each image to be recorded with a single exposure and reduces the time required for a full tomographic scan. These properties make this an interesting laboratory source for many tomographic imaging applications.

Imaging of bone is the oldest and most common form of medical x-ray imaging. However radiographs of bones do not typically reveal their microstructure, even though this is an important indicator in the diagnosis and treatment of diseases such as osteoporosis^1^, which is an increasingly widespread and costly disease due to the ageing of the human population. To determine the small scale structure of bone, three-dimensional imaging is required at high spatial resolution. X-ray computed tomography (CT) allows the 3D reconstruction of the interior of a sample by recording the x-ray transmission through it over many different projection angles^2^. Micro-computed tomography (*μ*CT)^3^,^4^, usually defined as CT with a spatial resolution of at least 100 *μ*m, has now emerged as the leading method for determining the internal microstructure of human bone^5^.

In commercial *μ*CT scanners, a 30–200 keV electron beam is focussed onto a high-*Z* anode to produce x-rays at a characteristic *K*_*α*_ energy along with a broad background of bremsstrahlung. The x-ray beam illuminates a sample to form an image via point-projection, since x-ray optics typically do not exist for these energies. Image contrast is generated by differential transmission *e*^−*μl*^ of x-rays due to the spatial variation in the mass absorption coefficient *μ*(*x*, *y*, *z*) in the sample of length *l*. As *μ* varies strongly with photon energy, the correct x-ray spectrum must be chosen to maximise image contrast, which can be dependent on the discrete electron beam anode materials available. For a fixed length beamline, higher spatial resolution can be achieved by focussing the electron beam more tightly to minimise the x-ray source size. However to prevent melting of the anode, this comes at the expense of reduced electron beam current and thus longer exposure time. The number of projection angles to record is also important; too few and the resolution of the reconstruction is degraded, too many and the total scan time and radiation dose become prohibitive.

Trabecular or cancellous bone is a type of bone with spongy form and tends to be affected more severely by osteo-degenerative diseases than denser bone types due to its high porosity^6^, leading to higher metabolic activity and consequent rate of bone turnover. Trabecular microarchitecture is an important indicator of bone strength^7^ and 2D histological and radiographic examination is insufficient to fully diagnose important details of the trabecular connectivity^8^. As such there is great medical interest in *μ*CT examination of the microstructure of trabecular bone samples, including 3D morphological characteristics^9^, mineral distribution^10^,^11^ and prevalence of micro-cracks^12^. However the imaging of trabecular bone presents significant challenges due to its high opacity and intricate internal structure. This makes trabecular bone an ideal test object for next generation light sources.

Synchrotron light sources have been trialled in this context^13^,^14^. Their high average brightness is helpful in two respects: first, the beam may be monochromated whilst retaining high flux; and secondly, the lower required exposure time allows more projections to be recorded in a given period, allowing a significant boost in speed of full 3D reconstruction^15^. Unfortunately synchrotron light sources driven *by conventional accelerators* will never become commonplace due to their prohibitive size and cost, and thus access to them for *μ*CT will remain limited.

Recently, synchrotron sources based on laser-wakefield accelerators (LWFA) have aroused great interest^16^,^17^. LWFA are promising cheap, compact sources of high-energy electron beams^18^,^19^,^20^,^21^,^22^. In a LWFA, a high-intensity short-pulse laser (*I* ≳ 10^19^ Wcm^−2^) is incident upon a gas, which instantly ionises to form a plasma. The radiation pressure of the laser pulse creates a single electron-depleted cavity (or ‘bubble’) behind it^23^. Plasma electrons trapped inside this cavity experience both longitudinal and radial focussing fields and so oscillate transversely whilst being accelerated forward. These so-called betatron oscillations cause the electrons to emit synchrotron-like radiation confined to a narrow cone in the forward direction (see Methods). The radiation spectrum is characterised by an energy *E*_*crit*_, close to the peak of the synchrotron spectrum. The betatron oscillation radius, *r*_*β*_, determines the x-ray source size and can be as low as 1 *μ*m^24^,^25^, comparable to or exceeding the state-of-the-art in conventional *μ*CT. Since the plasma is almost instantly replenishable, there is no equivalent anode-dependent limitation on source size and x-ray power.

The high *peak* brightness and small source size of laser-betatron x-ray beams have been used for high resolution x-ray imaging of insects^26^,^27^. However the typical beam energies *E*_*crit*_ ≲ 20 keV restricted imaging to quite transparent targets, and would be insufficient for most medically relevant applications such as high-resolution bone imaging. Here we demonstrate, by using wakefield acceleration at the near-GeV level, that we can produce the required x-ray spectrum and brightness for single-shot bone imaging whilst maintaining the advantageously small x-ray source-size.

In our experiment, the 300 TW Astra-Gemini laser pulse was focussed into a variable length helium gas cell to produce an electron beam by self-injection^19^ with energies up to 1.1 GeV and >100 pC total charge (see Methods), producing a co-propagating betatron x-ray beam-see Fig. 1a. A 7 mm diameter cylindrical section of human femoral trabecular bone was placed in the x-ray beam on a mount 70 cm from the exit of the gas cell. A point projection image was produced on an x-ray CCD camera a further 120 cm away, at a geometric magnification of 2.7.

The length of the gas cell could be adjusted to vary the output electron energy and the x-ray characteristics. The length was optimised so as to enhance the contrast in the resulting bone radiographs. Though the beam becomes brighter for longer lengths, the apparent source size increases due to the geometric smearing of the divergent x-ray beam being produced at different lengths within the gas cell^28^. The optimum was found for an electron beam with a peak energy of 720  ±  100 MeV produced from a 1.2 cm gas cell at density *n*_*e*_ = (2.9 ± 0.2) × 10^18^ cm^−3^ (see Methods). All quoted errors represent ± 1 standard deviation in a given quantity. Figure 1b displays a sample of electron spectra as recorded during the tomographic imaging run. The spectra were found to typically consist of a sharp quasi-monoenergetic peak and a broader low energy tail. The best-fit x-ray spectrum as recorded during the tomographic imaging is plotted in Fig. 1c. The x-ray beam divergence was found to be 10 × 20 mrad, exhibiting pointing fluctuations of ±2.1 × 1.5 mrad (horizontal×vertical). Each beam contained 1.3 ± 0.5 × 10^9^ photons above 1 keV. Under these conditions the FWHM x-ray source size was estimated to have an upper bound in the range of 2–3 *μ*m, consistent with the electron oscillation amplitude required to produce the observed spectrum, measurements (see Methods) and theoretical predictions.

Affixed to the sample rotation stage were two wires which rotated along with the sample and are visible in Fig. 2a. The axis of rotation can be determined by tracing the projected position of the ends of the wires as the stage rotates. A third fiducial was fixed independent of the stage allowing correction for the small shot-to-shot fluctuations in the position of the x-ray source. The transverse and longitudinal positions of the sample were chosen to achieve high magnification while keeping the entire sample in view over all possible rotation angles. A total of 235 projections were acquired.

## Results

**Figure 2:**
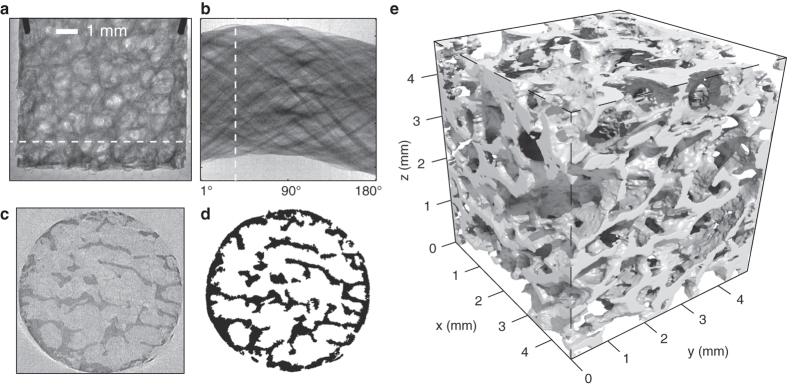
Tomographic reconstruction of trabecular bone sample: (**a)** A raw image of the bone sample recorded on the x-ray camera. (**b**) A sinogram of a particular row in the image, generated by stitching together 180 images of the same row taken at 1° intervals. (**c**) Application of the inverse Radon transform to the sinogram in (**b**) generates a 2D reconstruction of a one-pixel high horizontal slice of the sample. (**d**) Pixels are classified as bone (black) or vacuum (white) if their gray values are below or above the local mean. (**e**) Stacking together 1300 such slices generates a 3D voxel map of the bone sample. An isosurface marking the detailed structure of the bone surface is constructed, rendered using a ray-tracing method.

An exemplary radiograph is shown in Fig. 2a, along with the various steps towards 3D reconstruction of the bone surface in Fig. 2e (see Methods). The voxel size is 4.8 × 4.8 × 4.8 *μ*m^3^ as determined by the geometric magnification and limited by the pixel size of our detector. The intrinsic size of our x-ray source was smaller than a voxel and so effectively does not contribute to the final resolution. The resolution is instead dominated by the Poisson photon noise and the finite number of projections recorded. In the final reconstruction, structures with dimensions of 50 *μ*m or less are easily observed.

The low divergence of the source means the sample is illuminated by approximately parallel rays as in other synchrotron sources, simplifying the reconstruction algorithm. Though synchrotron-based *μ*CT operates at much higher flux and so can be much faster (completing a scan in as little as 0.5 s^29^), often they operate at repetition rates much higher than the detector frame rates. This can come at the expense of increased total dose that can lead to sample damage^30^. The laser based source can be better synchronised to detector response times. We estimate^31^ that the total dose received over the scanning period was 40 mGy, comparable to conventional imaging modalities^32^.

**Figure 3:**
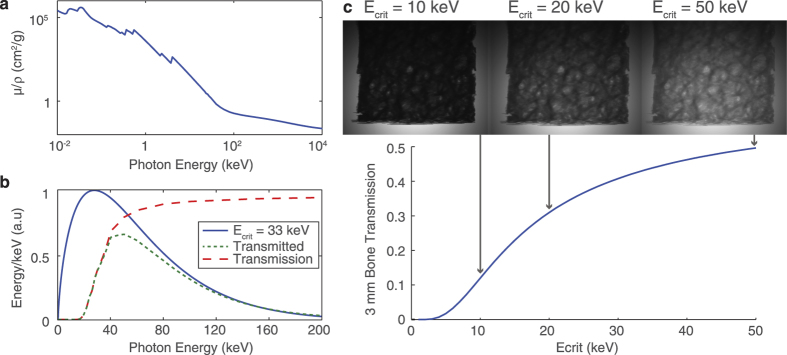
Synthetic images show which spectra are appropriate for tomography of the bone sample: (**a**) The mass attenuation coefficient of bone as a function of incident photon energy where the x-ray transmission is 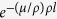 and *ρ* is the density and *l* the thickness of the bone. (**b**) The mass attenuation coefficient may be converted into a spectral transmission function, applied here to a spectrum of *E*_*crit*_ = 33 keV. The ratio between total integrated incident and transmitted energy gives an overall transmission factor. (**c**) The 3D reconstruction may be virtually illuminated with a synchrotron-like spectrum given the calculated total transmission factors and camera response function. Good image contrast is observed for approximately 15 keV < 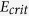 < 50 keV, but beyond this region the sample is either too opaque or too transparent.

The importance of being able to optimise *E*_*crit*_ is demonstrated in Fig. 3. Using the reconstructed structure and x-ray absorption characteristics of human bone (Fig. 3a), absorption contrast images were simulated for incident synchrotron spectra of varying *E*_*crit*_. An example of the spectral transmission is plotted in Fig. 3b. For *E*_*crit*_ = 10 keV, the sample would be almost completely opaque (Fig. 3c). For the most energetic beams generated in the present experiment, where *E*_*crit*_ = 55 keV, the sample becomes over 50% transparent at its thickest point and again image contrast is reduced. The images are optimised for intermediary *E*_*crit*_, in the range measured for the experimentally determined optimum contrast: *E*_*crit*_ = 33 ± 12 keV. This photon energy range could be readily accessed in the experiment, and the ability to easily vary the beam energy for thicker or thinner targets is a further attraction of this source.

## Discussion

**Figure 4:**
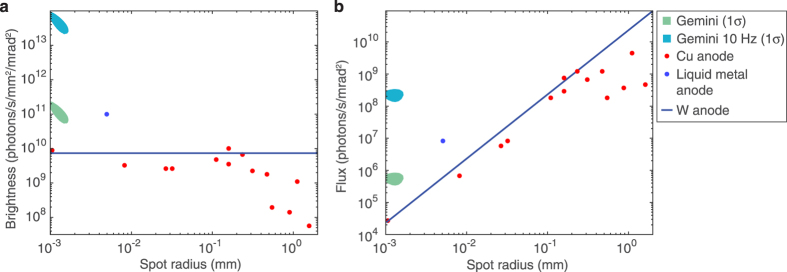
The brightness and flux of the laser-driven source at current and proposed repetition rates compare favourably to existing microfocus sources: (**a)** Given the measured fluctuation in photon number and source size, the 1*σ* contour of expected brightness is plotted in the brightness-spot size plane, plotted in units of photons per second per mm^2^ of source area per mrad^2^ of solid angle. Experimental data from microfocus sources are plotted as points, adapted from^37^,^38^. The solid line represents the simulated upper limit of a tungsten anode, using measured anode melting points^39^ and simulated spectra^40^. The number of photons is integrated over energies 10 keV < *E*_*γ*_ < 100 keV. (**b**) Accounting for the x-ray spot size, the brightness data are plotted as an average photon flux.

Since the source here is pulsed, the exposure time for each projection is effectively zero, and the limiting factor on the total scan time is the repetition rate of the laser. With the Gemini laser, operating presently at a shot every 40 seconds, the full high-resolution *μ*CT scan as shown here could be performed in 2 hours of laser time. Despite this relatively low repetition rate, the high collimation and photon number of the x-ray beam means that this is comparable to the time required for similar investigations with existing microfocus x-ray sources. This is demonstrated in Fig. 4 which shows the average photon flux and brightness of our source as compared to microfocus x-ray sources. The high photon energies required for bone tomography require these microfocus sources use high-Z anode materials, commonly tungsten, which result in a broad bremsstrahlung-dominated x-ray spectrum. In Fig. 4 we therefore integrate over photon energies of 10–100 keV (comprising 64% of the photons above 1 keV at *E*_*crit*_ = 33 keV) when comparing the x-ray sources. For the laser-driven source we measure an average brightness of 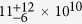 s^−1^mm^−2^mrad^−2^ and an average flux of 5.9 ± 2.4 × 10^5^ s^−1^mrad^−2^. However, given that the signal-to-noise level in some parts of the reconstruction can become low here, we suggest that for this type of bone sample a single-shot photon number of approximately 10^9^ should be regarded as a lower limit for reliable reconstruction using the filtered back projection method. The figure shows that our source is already competitive with existing microfocus x-ray sources, especially at the small source size required for high resolution imaging. If the Astra-Gemini laser were to operate at a repetition rate of 10 Hz, pumped by a diode-pumped system as is planned for the future^33^, the flux and brightness of this source would be increased by a factor of 400. As indicated in Fig. 4, it would then possess a unique combination of small source size and high average flux. 200–300 TW systems operating at 10 Hz are commercially available, and with such a system the scan described here could be completed in less than a minute if coupled to a suitably fast detector. Additionally, the resolution of the 3D reconstruction would benefit from the recording of more projection angles in a given time. Furthermore, scientists have already begun to dream about kHz operation of LWFA^34^, and consequently of this type of synchrotron source. Beyond considerations of average photon flux the very high *peak* flux of LWFA sources provides the high temporal resolution required for *in vivo* imaging, as the exposure time of each projection is only tens of femtoseconds. Temporal resolution may have benefits in imaging of bone structure, where it may become possible to study microcrack formation in real-time.

In summary, we have demonstrated that it is possible to use a LWFA to drive an x-ray source at the high photon energies required for the imaging of medically-relevant bone samples. The source size remains small at these high energies, and is sufficiently stable that we were able to perform micro-computed tomography in a reasonable time. We measured a photon flux which is already comparable to commercial microfocus sources, but note that the use of a high-repetition-rate laser will enable a dramatic increase in flux without compromising source size. Coupled to the proof-of-principle demonstrated here, the prospect of bringing laser-based *μ*CT sources to the laboratory seems bright.

## Methods

### Laser

The experiment was carried out at the Rutherford Appleton Laboratory, UK, on the Astra-Gemini laser facility. The laser wakefield acceleration pulse was linearly polarised with a central wavelength of 800 nm and a temporal FWHM of 40 ± 3 fs. 11.4 ± 0.4 J was delivered to the target and focussed with an *f*/20, 3 m focal length parabolic mirror to a slightly elliptical spot size of 25 × 32 *μ*m FWHM. The resulting peak intensity at vacuum focus was (1.8 ± 0.6) × 10^19^ Wcm^−2^, or a normalised vector potential of *a*_0_ = 3.0 ± 0.1. 27 ± 3% of the laser energy was contained within the FWHM contour of the focal spot.

### Gas target

A gas cell was used to confine helium gas to a defined length controlled by a set of linear motors. Fine control of plasma density was achieved by altering the pressure of the gas supply, and the timing of the gas entry valve with respect to the arrival of the laser pulse. The plasma electron density was recorded on every shot with transverse Mach-Zehnder interferometry. The gas cell parameters corresponding to the optimum x-ray beam for bone imaging were *n*_*e*_ = (2.9 ± 0.2) × 10^18^ cm^−3^ and length 1.2 cm.

### Sample reconstruction

With a rotation reference of 0° chosen arbitrarily, x-ray images were taken at 1° intervals from 1° to 180° and then at 12° intervals from 180° to 360°; the finely spaced set to perform the reconstruction with, and the coarser set to help determine the rotation axis. Before reconstruction each projection image was converted to a line integral of *μ* by dividing by the beam profile and taking the logarithm, and hot pixels removed with a selective median filter. Each row of the image is then analysed separately as a distinct slice of the bone sample, which may be denoted by a 2D function *f*(*x*,*y*), and a projection of this function at angle *θ* as *P*_*θ*_(*r*). In the two-step process of filtered back-projection^35^, the projections are first filtered to form


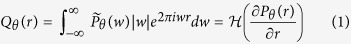


where 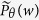 is the Fourier transform of *P*_*θ*_(*r*) and 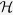 denotes the Hilbert transform. The filtered projections are then ‘smeared’ radially and summed to reconstruct *f*(*x*, *y*) as


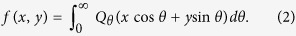


The reconstruction is classified pixel-by-pixel as either bone (black) or vacuum (white) in a process known as thresholding. The thresholded slices are stacked together to form a 3D array representing the bone sample, the cubic elements of which (voxels) have the same linear dimensions as a square camera pixel. By tracing and rendering the surface dividing bone from vacuum in the voxel array the structure of the bone is visualised in Fig. 2e. To ease the demands on the computational rendering, the surface visualisation was performed on a 5 × 5 × 5 binned dataset with a 24 *μ*m voxel size.

### X-ray beam characterisation

The on-axis energy spectrum of the radiation from an electron beam of energy *γ*_*e*_*m*_*e*_*c*^2^ has a wiggler-like shape^36^.


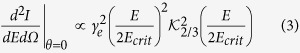


where *I* is the radiated energy, 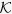 is a modified Bessel function of the second kind and the critical energy 
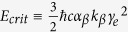
. Here, 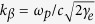 is the wavenumber associated with the betatron oscillation, *ω*_*p*_ is the plasma frequency and *α*_*β*_ = *γ*_*e*_*k*_*β*_*r*_*β*_ is the wiggler strength parameter. *E*_*crit*_ is close to the peak of the maximum of the synchrotron emission spectrum and hence characterises the typical photon energy of the distribution. Accounting for the dependencies of the various parameters, *E*_*crit*_ is found to scale approximately linearly with laser power and inverse plasma density^31^. The x-ray beam was characterised at different plasma densities, gas cell lengths, gas compositions and laser focussing positions. A 64-element filter array composed of various elemental metals with different *K*-edges was placed directly in front of the x-ray camera. Comparing the measured transmission through each filter with that expected from a synchrotron spectrum, the best-fit critical energy was obtained^17^. The beam profile was measured by optically imaging a CsI scintillator placed along the beam axis. The source size was measured by imaging a 1 mm-thick Si crystal at high magnification, and deconvolving the assumed Gaussian intensity profile from the recorded line spread function^17^.

### Data availability

The authors confirm that the all data used in this study are available without restriction. Data can be obtained by contacting plasma@imperial.ac.uk.

## Additional Information

**How to cite this article**: Cole, J. M. *et al*. Laser-wakefield accelerators as hard x-ray sources for 3D medical imaging of human bone. *Sci. Rep*. **5**, 13244; doi: 10.1038/srep13244 (2015).

## Figures and Tables

**Figure 1:**
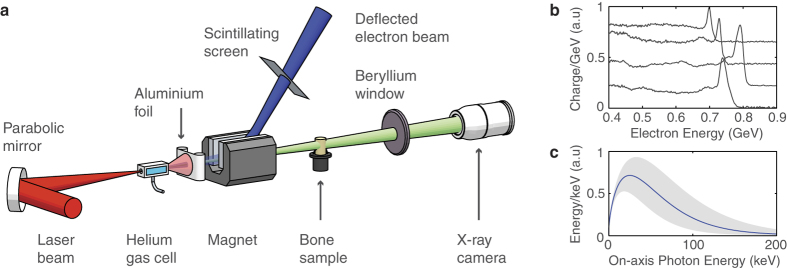
Laser wakefield x-ray imaging beamline: (**a)** Experimental layout (drawing produced by J. M. Cole, not to scale). The laser beam is focussed into a helium gas cell, producing high-energy electron and x-ray beams. The depleted laser pulse is blocked with a 25 *μ*m Al foil and the electron beam is swept away by a 1 T permanent magnet onto a scintillating LANEX screen, imaged into a CCD to measure the electron spectrum. The x-ray beam passes through the sample mounted on a stage with rotation and 3-axis translation motions at a distance of 70 cm, then out of a 180 *μ*m thick beryllium vacuum window. The detector is a CsI scintillator fibre-coupled to a 16-bit 2048 × 2048 cooled CCD array, which is a further 120 cm away. (**b)** A sample of typical electron beam spectra displaying a characteristic quasi-monoenergetic peak and broad low energy tail, separated vertically for visual clarity. (**c)** A best-fit incident synchrotron x-ray spectrum as recorded during the tomographic imaging (see Methods). The shaded area represents the uncertainty in the spectrum as determined by the electron energy fluctuations.
